# Supplementary material for: Label-Free Quantification Mass Spectrometry Identifies Protein Markers of Chemotherapy Response in High-Grade Serous Ovarian Cancer
Source: Cancers (Basel). 2023 Apr 6;15(7):2172. doi: 10.3390/cancers15072172 (PMC10093294; doi:10.3390/cancers15072172)
Supplement: Supplementary file 1 [file cancers-15-02172-s001.zip › cancers-2228604-Table S1.pdf]

Supplementary Table S1: Clinicopathological information for the patients used for the construction of TMA

Patient	First treatment	Grade	Category	Age at diagnosis [years]
1	Carboplatin / Paclitaxel	3	Complete Response	54
2	Carboplatin	3	Complete Response	79
3	Carboplatin / Paclitaxel	3	Complete Response	63
4	Carboplatin / Paclitaxel	3	Complete Response	76
5	Carboplatin/Paclitaxel	3	Complete Response	74
6	Carboplatin/Paclitaxel	3	Complete Response	59
7	Carboplatin/Paclitaxel	3	Complete Response	69
8	Carboplatin	3	Complete Response	69
9	Carboplatin/Paclitaxel	3	Complete Response	61
10	Carboplatin/Paclitaxel	3	Complete Response	43
11	Carboplatin	3	Complete Response	82
12	Carboplatin/Paclitaxel	3	Complete Response	49
13	Carboplatin/Paclitaxel	3	Complete Response	46
14	Carboplatin/Paclitaxel	3	Complete Response	51
15	Carboplatin/Paclitaxel	3	Complete Response	37
16	Carboplatin/Paclitaxel	3	Complete Response	64
17	Carboplatin	3	Complete Response	60
18	Carboplatin/Paclitaxel	3	Complete Response	59
19	Carboplatin	3	Complete Response	75
20	Carboplatin/Paclitaxel	2	Complete Response	59
21	Carboplatin	3	Complete Response	50
22	Carboplatin/Paclitaxel	3	Non-response/incomplete Response	78
23	Carboplatin / Paclitaxel / Avastin	3	Non-response/incomplete Response	66
24	Carboplatin/Paclitaxel	4	Non-response/incomplete Response	75
25	Carboplatin/Paclitaxel	3	Non-response/incomplete Response	61
26	Carboplatin/Paclitaxel	3	Non-response/incomplete Response	62
27	Carboplatin/Paclitaxel	3	Non-response/incomplete Response	44
28	Carboplatin/Paclitaxel	3	Non-response/incomplete Response	75
29	Carboplatin	3	Non-response/incomplete Response	78
30	Carboplatin	3	Unknown Response	79
31	Carboplatin/Paclitaxel	3	Unknown Response	60
